# Tumor necrosis factor-α suppresses the protein fractional synthesis rate of the small intestine stimulated by glutamine in rats

**DOI:** 10.3892/etm.2014.2129

**Published:** 2014-12-11

**Authors:** JIHONG ZHOU, SHENGXIAN FAN, YACHENG CAO, MINGFANG ZHU, YONG HAN, XUEYING CAO, YOUSHENG LI

**Affiliations:** 1 Department of Burns and Plastic Surgery, Jinling Hospital, Nanjing University School of Medicine, Nanjing, Jiangsu 210002, P.R. China; 2 Department of General Surgery, Jinling Hospital, Nanjing University School of Medicine, Nanjing, Jiangsu 210002, P.R. China; 3 Institute of Soil Science, Chinese Academy of Sciences, Nanjing, Jiangsu 210002, P.R. China

**Keywords:** ASCT2, fractional synthesis rate, glutamine, glutamine transporter, tumor necrosis factor-α

## Abstract

The objective of this study was to examine whether and how TNF-α affects glutamine-enhanced protein synthesis and the expression of the amino acid transporter ASCT2 in the small intestine at the mRNA and protein levels. A total of 30 male Sprague-Dawley rats were randomly assigned into three groups, namely the total parenteral nutrition (TPN; control), glutamine-treated (Gln), and glutamine- and tumor necrosis factor-α (TNF-α)-treated (TNF-α) groups. At 30 min prior to examination, all rats were mainlined with [L-^15^N]leucine. The concentration of TNF-α in plasma and of glutamine in plasma and the small intestine was measured. The fractional synthesis rate (FSR) of protein and the mRNA and protein expression levels of ASCT2 in the small intestine were assessed. The level of TNF-α was highest in the TNF-α group and the glutamine concentration was elevated to a greater extent in the TNF-α group than in the other two groups. However, the FSR of protein in the small intestine was significantly higher in the Gln group compared with that in the TNF-α group. The mRNA and protein expression levels of ASCT2 in the experimental groups were significantly higher that those in the control group, but did not differ significantly between the Gln and TNF-α groups. These results indicate that TNF-α may attenuate glutamine-stimulated protein synthesis in the small intestine in the early stage of sepsis in rats. The mechanism may be that TNF-α inhibits the function of the glutamine transporter in the uptake the glutamine into target cells for protein synthesis. This inhibition may occur at or following protein translation.

## Introduction

Sepsis is the leading cause of mortality in intensive care units around the world (1). The correct diagnosis and treatment of sepsis is complicated, but must be conducted quickly since a delayed approach increases the risk of mortality. However, following serious injury or illness in animal models of sepsis, the concentrations of glutamine, which is the most abundant free amino acid in the human body under normal conditions, in plasma and tissues fall sharply due to increased requirements by the inflammation-recruited immune cells (2–4). Low plasma glutamine concentration is associated with poor clinical outcome and increased risk of mortality (5). Studies have demonstrated that glutamine can effectively attenuate the rapid progression of sepsis that leads to multiple organ failure and eventually mortality (4,6–11). However, certain studies have disclosed that the effect of glutamine is not ideal in sepsis, especially in the early stage (12–14).

The plasma concentration of glutamine is maintained at a constant level and depends on the relative rate of net amino acid uptake and release by a number of organs. The movement of luminal amino acids across the intestinal brush border membrane is the initial step for transporting exogenous amino acids into systemic circulation. Several transport systems have been implicated in mediating the transport of neutral amino acids, including glutamine, in intestinal epithelial cells. Of these, the main contributor is the Na^+^-dependent neutral amino acid transport system Ala-, Ser- and Cys- preferring (ASC), which has established a transporter superfamily (15,16). System ASC transporters ASCT1 and ASCT2 are subfamilies that exhibit distinct substrate selectivity. ASCT1 transports Ala, Ser, Thr, Cys and Val, whereas ASCT2 has a broader substrate selectivity. ASCT2 also takes up glutamine with high affinity and accepts a wide range of other amino acids with longer side chains (asparagine, leucine, and isoleucine) (17). In sepsis, particularly in the early stage, the glutamine concentration in the small intestine is significantly decreased and its supplementation causes the arterial plasma concentration of glutamine to increase, yet no effects on the rate of protein synthesis in the small intestine have been observed (18). The mechanism remains unknown. Inflammatory mediators have an essential role in sepsis (19). It is therefore hypothesized that the elevating effect of glutamine on the protein synthesis rate may be attenuated by pro-inflammatory cytokines. The aim of this study was to investigate whether tumor necrosis factor-α (TNF-α), one of the major mediators of sepsis (19,20), inhibits glutamine-induced protein synthesis in the small intestine in a rat model of sepsis. Understanding the mechanism responsible for the lack of effectiveness of glutamine supplementation on protein synthesis may be beneficial to future therapeutic strategies for nutritional support in sepsis.

## Materials and methods

### Animals

A total of 30 male Sprague-Dawley (SD) rats, weighing 120–180 g (Shanghai Experimental Animal Center, The Chinese Academy of Sciences, Nanjing, China), were used. For 7 days prior to the experiments, the rats were housed in a temperature-, humidity-, and light-controlled environment on a 12 h light/12 h dark cycle and were allowed standard rat chow and water *ad libitum*. The standard rat chow contained (per 100 g): 50 g sucrose, 20 g casein-vitamin free, 15 g corn starch, 5 g powdered cellulose, 5 g corn oil, 3.5 g AIN-76A mineral mix, 1 g AIN-76 vitamin mix, 0.3 g DL-methionine, 0.2 g choline bitartrate, and 0.001 g ethoxyquin. The protocol and animal use were approved by the Animal Research Committee of Nanjing University (Nanjing, China). All procedures were carried out in accordance with the principles of the Guide for the Care and Use of Laboratory Animals (National Institutes of Health publication no. 85–23, revised 1985).

### Surgical procedures

Following an overnight fast, the rats were anesthetized with an intraperitoneal injection of ketamine (100 mg/kg body weight). The total parenteral nutrition (TPN) model was constructed, and a rotary transfusion apparatus was used for TPN infusion as described by Eizaguirre *et al* (21). Immediately following the surgery, all rats were maintained in individual metabolic cages and denied access to food but allowed free access to water to prevent dehydration and maintain hemodynamic homeostasis. Catheterized rats were connected to an infusion pump (Catalogue Celsite^®^; B. Braun Melsungen AG, Tuttlingen, Germany) and saline was infused at an initial rate of 5 ml/100 g body weight per hour.

### Experimental design

As the aim of this study was to investigate whether TNF-α inhibits the regulatory effect of glutamine on protein synthesis in the small intestine, it was first tested whether glutamine stimulated protein synthesis in the small intestine and then whether this stimulation was attenuated by TNF-α was examined. Thus, after 24 h of catheterization, the rats were randomly divided into the following three groups, each containing 10 rats: TPN (control), Gln (treated with glutamine) and TNF-α (treated with glutamine and TNF-α). The rats in the control group received TPN; the Gln group was given glutamine-enriched TPN, and the TNF-α group was received glutamine-enriched TPN followed by intravenously infused TNF-α for the last 24 h of the experiment. The parenteral nutrition solution was infused via the infusion pump at a constant rate of 1.5–2 ml/h. The TPN solutions were isonitrogenous and isoenergetic, containing 3.8 kJ/ml as a 60% carbohydrate and 40% lipid solution, representing ~502 kJ/kg/day and 0.75 g nitrogen/kg/day.

The basic formulations included amino acids (Novamin; Fresenius Kabi Pharmaceutical Co., Ltd., Beijing, China), glucose and lipids (Lipovenoes; Fresenius Kabi Pharmaceutical Co., Ltd.). The TPN mixture contained no glutamine, whereas the glutamine-enriched TPN mixture contained 0.15 g nitrogen per liter and was supplied by Dipeptiven (Glamin^®^, Fresenius Kabi AB, Uppsala, Sweden). The detailed compositions of the two solutions are shown in Table I.

The TPN mixture solutions were freshly prepared aseptically in a laminar flow hood each day. Nutrients were continuously infused for 3 days. Recombinant human TNF-α (PeproTech EC Ltd., London, UK) was diluted in 0.9% saline and infused intravenously for the final 24 h at a rate of 5 μg/kg/h, in accordance with a previous study (22). Thirty minutes prior to sampling, all rats were injected with [L-^15^N leucine (1 mmol/kg; Cambridge Isotope Laboratories, Inc., Andover, MA, USA).

### Sample analysis

The rats were sacrificed by decapitation after 72 h of TPN. Blood was collected into lithium heparin tubes and centrifuged at 3,000 × g at 4°C for 10 min. The plasma was decanted and stored at −70°C until analysis. The small intestine was rapidly dissected, and the dissected small intestine was plunged into an ice-water slurry (0°C–4°C), at which time it was assumed that all metabolic processes including protein synthesis had ceased or were negligible. Samples of the small intestine were then weighed, flash-frozen in liquid nitrogen, and subsequently stored at −70°C until analysis.

### TNF-α measurement

The plasma level of TNF-α was analyzed with a sandwich enzyme-linked immunosorbent assay from PeproTech EC Ltd. and performed according to the manufacturer’s instructions.

### Glutamine concentration in plasma and small intestine

The glutamine concentration in plasma and tissue from the small intestine, extracted from a homogenate, were measured using high-performance liquid chromatography, as described previously (23).

### Protein synthesis rate

Rates of protein synthesis were measured based on a ‘flooding dose’ technique described previously by Garlick *et al* (24). The small intestine was divided into two sections of similar weight, prior to freezing, each section weighing ~50 mg. Subsequently, protein analysis was performed on one section only, with the other section being used for the glutamine assay.

The sample of intestine was homogenized and then precipitated in 10 ml 0.2 mol/l protein perchloric acid. Following centrifugation (2,000 × g, 4°C, 15 min), the supernatant was decanted for subsequent neutralization and measurement of the enrichment of free amino acids (E_F_; see below). The protein pellets were washed twice in 10–12 ml 0.2 mol/l perchloric acid followed by centrifugation (2,000 × g, 4°C, 15 min) to remove contaminating free leucine. The pellets were then digested in 10 ml 0.3 mol/l NaOH at 37°C for 1 h, re-precipitating with 4 mol/l perchloric acid to further disrupt any cellular compartments that still contained free leucine. The pellets were then washed eight times with 0.2 mol/l perchloric acid and hydrolyzed in 3.0 ml 6 mol/l HCl for 24 h at 105°C. The hydrolysates were vacuum-dried. Dried residues were then suspended in 3.0 ml sodium citrate buffer (pH 6.3; 1.5 mol/l), and samples were stored at −4°C until processed for measurement of the enrichment of protein-bound leucine (E_B_) (25).

The fractional synthesis rate (FSR) of protein, defined as the percentage of tissue protein renewed by synthesis each day, was calculated by the following equation (26):

$$FSR=\frac{E_{B(t)}-E_{B(0)}}{\int_{0}^{t}E_{F(t)}dt}$$

E_B_ is the enrichment of ^15^N of protein-bound leucine, E_F_ is the enrichment of tissue-free leucine, and t is the period from injection of the isotope to the immersion of the tissue into iced water. The enrichment of ^15^N of protein-bound and tissue-free leucine was detected by mass spectrometry (Finnigan MAT; Thermo Fisher Scientific, San Jose, CA, USA).

### ASCT2 mRNA determination by reverse transcription quantitative polymerase chain reaction (RT-q)PCR

Total RNA was extracted from each 0.5 ml purified sample using TRIzol (Invitrogen Life Technologies, Carlsbad, CA, USA), and stored in 1 ml dehydrated alcohol at −80°C. The specific PCR primers and fluorescently-labeled probes that were used are listed in Table II. The total RNA from each sample was reverse-transcribed into first-strand cDNA in a 20-μl reaction system (RT buffer 10 μl, 100 pmol/μl primers 1 μl, RT-mix 1 μl, RNA 5 μl and deionized DEPC water 13 μl) under the following conditions: denaturation at 25°C for 10 min, annealing at 40°C for 60 min, and synthesis at 70°C for 10 min, for 40 cycles. qPCR was performed using a sequence detector system (FTC-2000; Funglyn Biotech Corporation, Toronto, Canada) with specific individual primer pairs and fluorescently-labeled probe in 50 μl reaction mixture (PCR buffer 25 μl, 25 pmol/μl primers 0.6 μl × 2, probe 0.4 μl, cDNA 1 μl, deionized DEPC water 22.4 μl) using the following conditions: 94°C for 4 min, annealing at 94°C for 30 sec, and synthesis at 60°C for 30 sec, for 40 cycles. The progress of the PCR amplification was monitored in real-time by fluorescent measurement during each cycle, and the relative concentration of the target gene was quantified with GAPDH. The analysis of PCR results and calculation were performed using Rotor-Gene software (version 6; Corbett Robotics Inc., Qiagen, San Francisco, CA, USA) and the control levels were set at 1. All procedures were performed at least three times.

### ASCT2 protein determination by western blotting

Protein samples (5 μg) were electrophoretically separated on SDS polyacrylamide gel and transferred to a polyvinylidene difluoride membrane that was blocked with blocking solution (5% w/v skimmed milk) at room temperature for 2 h. The membrane was then incubated overnight with the goat anti-mouse polyclonal antibody to ASCT2 (dilution 1:500; Santa Cruz Biotechnology, Inc., Santa Cruz, CA, USA) diluted in Tris-buffered saline with 0.1% Tween 20 (TBST). Then, the membrane was incubated with donkey anti-sheep IgG-HRP antibody (dilution 1:10,000; Santa Cruz Biotechnology, Inc.) at room temperature for 1 h and was treated with the enhanced chemiluminescence western blot (ECL-WB) assay (Perkin Elmer, Inc., Waltham, MA, USA). Semiquantitative assessment of bands was performed densitometrically using Quantity One software (Bio-Rad, Hercules, CA, USA). The detection and assessment procedure were repeated at least three times.

### Statistical analysis

Data are presented as the mean ± standard error of the mean. One way analysis of variance followed by the Student-Newman-Keuls test was used to assess the means among multi-groups while the Dunnett’s T3 test was used to assess pairwise multiple comparisons. Statistical Package for Social Sciences (SPSS, Inc., Chicago, IL, USA) version 11.0 was used for data analysis. Statistical significance was accepted at the P<0.01 level.

## Results

### Plasma TNF-α level

The plasma levels of TNF-α in the three groups are presented in Table III. Following the continuous infusion of TNF-α for 24 h, the TNF-α levels in the plasma were significantly elevated in the TNF-α group and were significantly higher than those in the other two groups (P<0.01). No statistically significant difference in TNF-α level was identified between the TPN (control) and Gln groups. Thus, the infusion protocol elevated the circulating TNF-α concentration to a high level.

### Plasma glutamine level

The plasma levels of glutamine are also presented in Table III. The plasma glutamine level was lowest in the TPN group, and increased markedly following treatment with glutamine and TNF-α, and was the highest in the TNF-α group. There was a significant difference among the three groups (P<0.01). Supplementation with a combination of glutamine and TNF-α significantly increased the free glutamine concentration in plasma.

### Small intestine glutamine level

The concentration of glutamine in the small intestine is shown in Table III. An increase in glutamine concentration was observed in the small intestine after 24 h of intravenous infusion with TNF-α compared with that in the TPN group (P<0.01). The maximal increase of glutamine concentration was observed in the group treated with a combination of TNF-α and Gln (P<0.01 compared with the Gln group). Thus, the combination of TNF-α and glutamine increased the free glutamine level in the small intestine.

### Small intestine protein FSR

As shown in Table III, there was a significant increase in the FSR of protein in the small intestine in the Gln group. The difference among the three groups was significant (P<0.01). The protein FSR was higher in the Gln group than in the TPN group, and it may be concluded that glutamine is able to increase protein synthesis in the small intestine. However, the effect of glutamine on protein synthesis was not observed in TNF-α group. Following treatment with TNF-α, the protein FSR of the small intestine in the TNF-α group was markedly decreased compared with that in the Gln group. Thus, TNF-α attenuated the stimulative effect of glutamine on protein synthesis in the small intestine of rats.

### mRNA expression level of ASCT2

**Figure 1:**
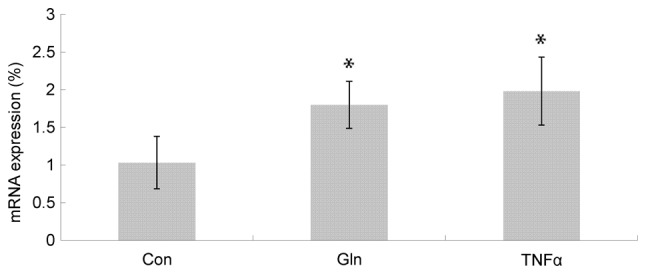
mRNA expression level of ASCT2 in the small intestine. The mRNA expression levels of ASCT2 in the experimental groups were significantly higher than that in the control (Con) group, and the difference between the Gln and TNF-α groups was not significant. All data are presented as the mean ± SEM. Con, total parenteral nutrition (TPN) group (n=10), supplied with TPN; Gln, glutamine-treated group (n=10), supplied with glutamine-enriched TPN; TNF-α, TNF-α and glutamine group (n=10), supplied with glutamine-enriched TPN and treated with TNF-α. ^*^P<0.01, compared with the control group.

As shown in Fig. 1, the mRNA expression levels of ASCT2 in the experimental groups were significantly higher than that in the TPN group (P<0.01). Although the mRNA expression level of ASCT2 was higher in the TNF-α group than that in Gln group, the difference between these two groups was not significant.

### Effect of TNF-α on the protein expression level of ASCT2

**Figure 2:**
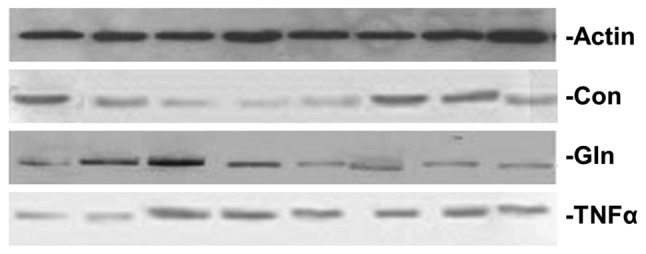
Protein expression of ASCT2 in the small intestine. Con, total parenteral nutrition (TPN) group, supplied with TPN; Gln, glutamine-treated group, supplied with glutamine-enriched TPN; TNF-α, TNF-α and glutamine group (n=10), supplied with glutamine-enriched TPN and treated with TNF-α.

**Figure 3:**
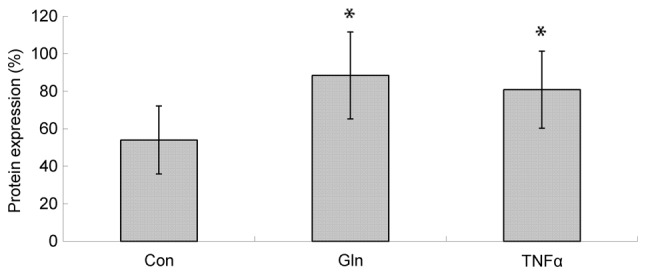
Protein expression of ASCT2 in the small intestine. The protein expression levels of ASCT2 in the experimental groups were significantly higher than that in the control (Con) group, and the level in the Gln group was slightly higher than that in the TNF-α group; however, the difference between the Gln and TNF-α groups was not significant. All data are presented as mean ± SEM. Con, total parenteral nutrition (TPN) group (n=10), supplied with TPN; Gln, glutamine-treated group (n=10), supplied with glutamine-enriched TPN; TNF-α, TNF-α and glutamine group (n=10), supplied with glutamine-enriched TPN and treated with TNF-α. ^*^P<0.01 compared with the Con group.

The protein expression of ASCT2 was identified in all groups (Fig. 2). Among the three groups, the protein expression levels of ASCT2 in the Gln and TNF-α groups were significantly higher than those in the control (Con) group (P<0.01), and were slightly higher in the Gln group than in the TNF-α group. However, the difference between the Gln and TNF-α groups was not statistically significant (Fig. 3).

## Discussion

Sepsis has become a major worldwide health problem due to the continual increase of its incidence, as well as its high mortality rate (27–29). Therefore, the prevention and treatment of sepsis has become an important focus in medical research. Previous studies have disclosed that glutamine supplementation in sepsis is not necessarily effective in increasing protein synthesis in the skeletal muscle of rats (30,31,32). Other studies have shown that in endotoxin-treated rats or rats with septic abscesses, parenteral supplementation with glutamine could only increase muscle glutamine levels, not muscle protein synthesis (32,33).

In the present study, the changes in rats induced by an intravenous injection of TNF-α were investigated. The results showed the plasma TNF-α rose to a high level and was similar to that observed in sepsis (34). The present study showed that a combination of TNF-α and glutamine increased the free glutamine concentration, and TNF-α increased the free glutamine concentration in plasma and the small intestine significantly. Previous simulation experiments have demonstrated that in the early stage of sepsis, the concentration of free glutamine is increased, not reduced (35). Similar to another study (36), the present study showed that glutamine could indeed promote protein synthesis, but that the function of glutamine could be inhibited by TNF-α.

Therefore, the high concentration of extracellular glutamine fails to be utilized well under infection conditions. As an amino acid, the process of transmembrane transport depends on the normal structure and function of glutamine transporters. The first glutamine transporter was isolated more than 40 years ago, and large numbers of transporters have now been isolated (37–40). ASCT2 is a transporter of the Ala-, Ser- and Cys-preferring (ASC) system. It has a high affinity for glutamine transportation, and is distributed widely in all kinds of cells (41–45). There have been numerous studies on ASCT2 with gradually advancing methods (17,46). The failure of a high concentration of extracellular glutamine to exert effects may occur as a result of the limitation of transporter function.

The present study also demonstrated that TNF-α had a modest promoting effect on the expression of ASCT2 mRNA, which indicates the suppressive effect of TNF-α did not occur at the transcription level. There are two major methods by which protein function is regulated: one is the alteration of expression while the other is conformational change at the functional site. The present study revealed that although TNF-α suppressed the expression of the ASCT2 glutamine transporter protein, the magnitude of the reduction was not significant, which indicated that the suppression of transporter function may be accompanied by structural changes to the transporter. However, the molecular mechanism of the effect of TNF-α on glutamine utilization remains unclear.

In conclusion, the present study suggests that TNF-α attenuates the glutamine-induced stimulation of protein synthesis in the small intestine. The mechanism may be that TNF-α inhibited the function of glutamine transporters in the uptake the glutamine into target cells for protein synthesis. This inhibition may occur at or after protein translation. However, additional studies on whether TNF-α affects the structure of glutamine transporters and on how TNF-α affects the structure of glutamine transporters are required.

## Figures and Tables

**Table I:** Compositions of amino acid solutions.

Amino acids	TPN (g/l)	Gln (g/l)
Alanine	12.2	9.8
Arginine	8.4	6.7
Aspartic acid	2.5	2.0
Cystine	0.2	0.2
Glutamic acid	4.2	3.3
Glycine	5.9	4.7
Histidine	5.0	4.0
Isoleucine	4.2	3.4
Leucine	5.9	4.7
Lysine	9.5	7.6
Methionine	4.2	3.4
Phenylalanine	5.9	4.7
Proline	5.0	4.0
Serine	3.4	2.7
Threonine	4.2	3.4
Tryptophan	1.4	1.1
Tyrosine	0.2	0.2
Valine	5.5	4.4
L-glutamine	-	15.9
Total amino acids	85.0	88.0
Total nitrogen	14.0	14.0

TPN, total parenteral nutrition. Gln is glutamine-enriched TPN. Composition of TPN: 1 l TPN provides 3,766 kJ and 7.5 g of nitrogen (kJ/nitrogen=502).

**Table II:** Sequences of qPCR primers and probes.

Primer/probe	Sequence
Primer	F: 5′-GCAGCCTAGACCTGGGATCAC-3′ R: 5′-GCGGTCTTTGATTCCCTGAA-3′
Probe	Fam - ATCTTGGGTTCCCGGAGCCAG ACAT - TAMRA

qPCR, quantitative polymerase chain reaction; F, forward; R, reverse; Fam, carboxyfluorescein; TAMRA, tetramethylrhodamine.

**Table III:** Plasma TNF-α level, plasma and small intestine glutamine levels, and the fractional synthesis rate of protein in the small intestine.

Variable	Con (n=10)	Gln (n=10)	TNF-α (n=10)
Plasma			
TNF-α (pg/ml)	7.903±2.119	5.442±1.926	1673.767±108.774^b^
Gln (μmol/l)	376.766±33.045	403.516±26.759^a^	440.816±44.434^b^
Small intestine			
Gln (μmol/l)	0.165±0.010	0.203±0.008^a^	0.428±0.012^b^
FSR (% per day)	4.005±0.020	4.669±0.040^a^	4.463±0.040^c^

The levels of tumor necrosis factor-α (TNF-α) and glutamine (Gln) were determined by an enzyme-linked immunosorbent assay and high-performance liquid chromatography, respectively. The fractional synthesis rate (FSR) of protein was determined using a stable isotope, as described in Materials and methods. Data are expressed as mean ± SEM. Con, total parenteral nutrition (TPN) group (n=10), supplied with TPN; Gln, glutamine-treated group (n=10), supplied with glutamine-enriched TPN; TNF-α, TNF-α and glutamine group (n=10), supplied with glutamine-enriched TPN and treated with TNF-α.

a P<0.01, vs. the TPN group,

b P<0.01, vs. the Gln group and

c P<0.05 vs. the Gln group, each as determined by analysis of variance followed by the Student-Newman-Keuls test.
